# The Treatment of Uterine Fibroids

**Published:** 1902-06

**Authors:** Virgil O. Hardon

**Affiliations:** Atlanta, Georgia


ATLANTA

### Journal-Record of Medicine.

**Successor to Atlanta Medical and Surgical Journal, Established 1855, and Southern Medical Record, Established 1870.**

VOL. IV. JUNE, 1902. No. 3.

BERNARD WOLFF, M.D., EDITOR, Nos. 319–20 Prudential.

Published Monthly.

M. B. HUTCHINS, M.D., BUSINESS MANAGER, No. 64 Marietta St.

### *ORIGINAL COMMUNICATIONS.*

### THE TREATMENT OF UTERINE FIBROIDS.*

BY VIRGIL O. HARDON, M.D.,

ATLANTA, GEORGIA.

Probably in no department of medical practice have greater changes occurred during the last decade than in the treatment of uterine fibroids. The time is within the recollection of us all when a fibroid tumor of the uterus was regarded as a matter of comparatively trivial importance, to be treated only by palliative measures, or in a large proportion of cases to be treated not at all. The patient was usually reassured by the statement that as soon as the menopause should occur the symptoms would cease spontaneously and no further trouble would exist. When symptoms arose of sufficient severity to require immediate attention, the treatment was tentative in character and was based upon the mistaken idea that the tumor was not a pathological growth, and that, therefore, the symptoms produced by it were merely incidental and might safely

* Read before the Georgia Medical Association, Savannah, April, 1902.

be ignored with reference to ultimate results. Since the most prominent symptom of uterine fibroids is usually hemorrhage from the womb, it was deemed only necessary to keep the loss of blood within the limits of safety and wait for nature to accomplish the expected cure.

The means adopted for carrying out this purpose were various in character, and their very variety furnishes sufficient evidence that all were unsatisfactory in their results. By some practitioners the womb was curetted upon the theory that the hemorrhage was due to a chronic endometritis produced mechanically by the presence of the tumor in the uterine wall. By others the cervix was dilated in an empirical way without recognition of the fact that such dilatation produced only a temporary contraction of the body of the womb whereby the hemorrhage was checked merely for the time being. Still others, following the teaching of the great Apostoli, sought by electrolysis to produce such structural changes in the tumor as would permit of its absorption by the pelvic lymphatics and blood-vessels. This mode of treatment was the cause of a considerable number of deaths from peritonitis, while in many other cases it was entirely devoid of results, beneficial or otherwise. In the hands of more timid physicians drugs were used to control hemorrhage, and hence ergot and hydrastis have played a prominent part in the treatment of these cases. Those of us whose memories can extend back through a quarter of a century can recall the vogue enjoyed by the injection of ergotine into the substance of the tumor, and I myself am cognizant of at least one death resulting from this crude and barbarous method. When all these means failed, as they usually did fail sooner or later, resort was had to the more radical treatment by oophorectomy, with the expectation of inducing a premature menopause which would control all the symptoms. These various methods of treatment were based upon the erroneous idea, once universally taught, that a fibroid tumor was practically nothing more than an hypertrophy of normal uterine tissue, and, therefore, possessed no more significance, *per se*, than would an hypertrophy of a voluntary muscle from continuous exercise. Modern research has demonstrated that this view of uterine fibroids is entirely incorrect.

The symptoms produced by uterine fibroids are of three kinds: 1st, hemorrhage, probably due to mechanical irritation of the womb from the presence of the fibroid; 2d, pain, due to pressure of the tumor upon the uterine structures or upon surrounding parts; 3d, interference with the functions of neighboring organs, especially the bladder and the rectum. It is true that there are some exceptional cases in which no symptoms are present. But even though at some certain period in the history there may be entire absence of symptoms, it seldom happens that this immunity continues throughout life. Since fibroids have an inherent tendency to continually increase in size, the time will almost inevitably arrive, sooner or later, when one or more of these symptoms will become so urgent as to make treatment imperative. In a large majority of the cases in which I have operated for fibroids of the uterus, I have found the tubes and ovaries the seat of so extensive disease as to constitute a sufficient reason for their removal. The tubal and ovarian disease in such cases appeared to be secondary to the uterine tumor, since in most instances there had been no evidence of such disease before the tumor was discovered. The tendency of fibroids to undergo sarcomatous degeneration is well recognized at the present day. If, in addition to these facts, it be conceded that the removal of these tumors can be accomplished much more easily and safely when they are small than when they have reached such a size as to produce pressure upon neighboring organs and to seriously impair the general health, a sufficiency of reasons will have been established why the radical treatment by removal is to be preferred to the palliative measures mentioned in the beginning of this paper.

In discussing the advisability of operative treatment for uterine fibroids, the first question which naturally arises relates to the prognosis in cases which are not subjected to operation. The statement found in some of the older literature that "no one ever dies of a fibroid tumor" has been too often disproved by clinical experience to require refutation at the present day. Deaths have occurred from hemorrhage, peritonitis, suppuration, sloughing of the tumor, and sarcomatous degeneration, as well as from general impairment of health which rendered the patient an easy prey to intercurrent affections. Even though death may not result, the patient's life in the majority of cases becomes a burden from chronic invalidism or from continuous pain. The prognosis, therefore, is not a cheerful one. There is a modicum of truth in the old idea that the menopause exerts a favorable influence upon such tumors. But the cases in which a fibroid disappears or diminishes by atrophy, or even ceases to grow, form but a small minority. Moreover the presence of the tumor causes the menopause to be delayed for several years beyond the normal period. Palliative treatment, though skilfully and continuously applied, gives only temporary results, and recurrence of the symptoms is usually inevitable.

A second question is, does removal of the tumor effect a cure? Since fibroids of the womb are confined to that organ and have no tendency to invade neighboring parts, it requires no argument to prove that removal of the womb means the removal of the original disease. But since in nearly all cases the ovaries and tubes will have become secondarily involved, it is necessary to remove the appendages with the uterus, in order to effect a cure with certainty and completeness. By such removal all of the disease, both primary and secondary, will have been eradicated, and there will be no possibility of recurrence. The prognosis therefore admits of no doubt, provided the patient survives the operation.

This brings up still another question bearing upon the subject. What mortality may be expected to follow the operation for the removal of fibroid tumors? This point can be determined only by the experience of individual operators. Of forty-three patients upon whom I have operated, three have died. These figures, however, fail to represent the normal mortality in operations undertaken early, before the patient has been reduced to an unfavorable condition by delay. In one of my fatal cases, the tumor had attained to an enormous size, reaching the ensiform cartilage. The functions of the heart were disturbed by pressure, the tumor had contracted adhesions to nearly all the abdominal organs, and the patient was in as unfavorable condition for operation as could possibly be imagined. In another fatal case hysterectomy had been advised while the patient was in fairly good condition, but it was not until eight months later, when she had become bedridden and death was impending, that she consented to the operation. In my third fatal case, the patient was suffering from goitre, and while apparently convalescent from the operation, died on the eighth day from heart complications. If the rule of early operation could have been applied to the first and second of these cases, it is probable that they would now be recorded as recoveries instead of deaths. In operations undertaken while the tumor is small and the patient in good condition it may be confidently expected that the mortality will not exceed 2 or 3 per cent. The time wasted in palliative treatment is usually the cause of the delay which carries the mortality to 6 or 8 per cent. Under favorable circumstances hysterectomy for fibroid tumor in skilled hands is no more hazardous than other abdominal operations. When the tumor is small it does not contract adhesions, it does not give rise to serious inflammatory complications, and the recovery from operation is usually rapid and uneventful.

There is still another point which requires consideration. It is a well-recognized and unquestionable fact that a certain proportion of fibroid tumors give rise to no symptoms whatever. How large this proportion may be it is impossible to say, since many such cases must obviously escape recognition. If they are discovered at all, it is only by accident, unless they have reached such a size as to impair the symmetry of the patient's figure or to be felt by her as a "lump" in the abdomen. It would be obviously absurd to maintain that tumors which give rise to no trouble should be removed by abdominal operation. But it not infrequently happens that the embarrassment caused by the enlargement of the abdomen, or the mental distress arising from a knowledge of the existence of the tumor, leads the patient to seek medical advice. In such cases the question of operation must be decided by the extent to which the comfort and happiness of the patient are compromised. If, however, the patient is unconscious of the existence of the tumor, it may safely be ignored for the time being. It must be borne in mind, however, that the tendency to continuous growth renders it highly probable that at some subsequent period symptoms will develop which will call for active treatment. The fact must also be considered that small tumors can be removed with much greater facility and safety than those which have attained to a considerable size. I am convinced that if the rule which is now universally applied to ovarian cysts, namely, that they should be removed as soon as discovered, could be applied to fibroid tumors, the statistics of hysterectomy would show nearly, if not quite, as small a mortality as those of ovariotomy. In my operations for ovarian cysts I have had, up to the present time, 100 per cent. of recoveries. The list includes dermoid, papillomatous, parovarian and simple cysts. If my operations for fibroid tumors had been undertaken as early as have the majority of those for ovarian tumors, the results would probably have been equally favorable, since the deaths which have occurred have been due to complications which are not usually found with small tumors. Hence, in cases where innocuous fibroids are found, the patient should be kept under observation and operation should be advised as soon as symptoms shall have developed.

Finally, when it has been determined that operation is indicated, a decision must be made between myomectomy and hysterectomy. If the tumor is pedunculated upon either the internal or the external surface of the womb, so that it has become a polypus, myomectomy is to be preferred. If it were possible in any given case to know that an intramural tumor was single and that no other foci of degeneration existed in the womb, myomectomy would still be the operation of choice. But such knowledge is impossible and, as a matter of fact, single foci rarely, if ever, exist. If one tumor is removed by myomectomy, the remaining foci are usually stimulated to greater activity, so that the original condition may be reproduced and a second operation may become necessary. Statistics show that the operation of myomectomy has as large a mortality as that of hysterectomy. The only advantage that can be claimed for myomectomy is that it does not destroy the child-bearing capacity of the woman. But this claim loses much of its weight in view of the fact that pregnancy seldom occurs in a fibromatous uterus. It appears to me an ill-judged conservatism that would dictate the retention of a diseased organ which has become functionally useless. For these reasons I would prefer hysterectomy to myomectomy, except in those cases in which the tumor has become pedunculated.

As a deduction from the views expressed in this paper, I would lay down two propositions:

1st. Small fibroid tumors which give rise to no symptoms, physical or mental, may be ignored for the time being.

2d. Fibroid tumors which give rise to symptoms, or which cause mental distress from disfigurement or from apprehension, should be removed as soon as the patient can be gotten into the best possible physical condition for operation.

Hysterectomy for fibroid tumors is not an operation for the novice. He who undertakes it must be prepared to cope with any complication, from the closure of an accidental opening into the bladder to the transplantation of a divided ureter or the resection of injured intestine. It is an operation which requires skill, experience, fertility of resource, thorough knowledge of anatomy, and complete preparation for any emergency. But when properly and skilfully done, there is no capital operation in surgery which yields more satisfactory results.

Ten years ago, in a paper read before the American Gynecological Society, Gordon of Maine, advocated essentially the same views that are expressed in this paper. But he was ten years in advance of his time and the medical world stood aghast at his proposition to remove all uterine fibroids as soon as they were discovered. But those men who have had the largest opportunities to study this class of cases have gradually come to adopt his views, and the time is near at hand when early removal will be recognized as the method of choice in the treatment of uterine fibroids.

